# A Generalized Radiation Model for Human Mobility: Spatial Scale, Searching Direction and Trip Constraint

**DOI:** 10.1371/journal.pone.0143500

**Published:** 2015-11-24

**Authors:** Chaogui Kang, Yu Liu, Diansheng Guo, Kun Qin

**Affiliations:** 1 School of Remote Sensing and Information Engineering, Wuhan University, Wuhan, Hubei, China; 2 Institute of Remote Sensing and Geographical Information Systems, Peking University, Beijing, China; 3 Department of Geography, University of South Carolina, Columbia, South Carolina, United States of America; 4 Collaborative Innovation Center of Geospatial Technology, Wuhan University, Wuhan, Hubei, China; Peking UIniversity, CHINA

## Abstract

We generalized the recently introduced “radiation model”, as an analog to the generalization of the classic “gravity model”, to consolidate its nature of universality for modeling diverse mobility systems. By imposing the appropriate scaling exponent *λ*, normalization factor *κ* and system constraints including searching direction and trip OD constraint, the generalized radiation model accurately captures real human movements in various scenarios and spatial scales, including two different countries and four different cities. Our analytical results also indicated that the generalized radiation model outperformed alternative mobility models in various empirical analyses.

## Introduction

The quantitative modeling of human movements has long-standing implications in fields like transportation, epidemiology, and urban planning. For instance, road traffic condition is remarkably predictable if characteristics of individuals’ travel behaviors, such as origin-destination distribution, road topology and mode of transportation, are efficiently incorporated [1, 2]. Epidemics are highly traceable if their transmission processes, including sources and sinks of infections, and suscepts’ social contacts, are timely captured [3]. As for urban settings, understanding individuals’ daily travel activities has enabled rational facility allocation [4], efficient zoning amendment [5], personalized venue recommendation [6], and to list a few. In particular, the emerging big (geo-) data has been boosting mobility related studies and making significant progresses in the field.

To date, a rich body of theoretical models for bridging mobility, physical distance and the effect of intervening opportunities have been developed in existing literature. Among them, gravity based models [7, 8] have been favored by practitioners due to its self-explanatory form and computational ease. Whereas, abundant statistical evidences have shown advantages of the concept of intervening opportunities [9] at explaining a broad range of mobility data. Recently, a new model called the “radiation model” [10] originating from diffusion dynamics earns its prevalence on the grounds of its simple form and parameter-free property. Along with other newly-proposed models including rank-based gravity model [11] and population-weighted opportunities model [12], they are inherently built upon Stouffer’s framework of intervening opportunities.

Despite the prospect of the radiation model, concerns on its “universality” have limited the applicability of the model to diverse mobility systems. Nevertheless, even if the radiation model in general gives competitive results, researchers realized that certain elements with substantial importance like spatial scale and heterogeneity are overlooked in the model [13]. Particularly, much literature argued that abilities of the radiation model are largely limited at the city level [12, 14]. They reported their empirical observations of human mobility in a couple of cities, including New York, London, Beijing, Chicago, Seattle, Shenzhen and Abidjan, and attributed the model failures to different reasons. For example, in intra-urban scenarios, mobility trips are thought less dependent on physical distance but more on the accessibility of resources satisfying the objective of the trip. In other words, individuals in an urban area differ from random walkers in exploring physical space because of the motivations driving their mobility. The parameter-free nature of the radiation model, unfortunately, largely limits its capability of quantifying the diverse motivations that initiate individual human mobility. The dilemma of “simplicity versus diversity” hence significantly challenged the radiation model, as shown in many empirical studies.

In this research, we generalize the radiation model to overcome the aforementioned limits while maintaining its nature of universality. First and foremost, we introduce a scaling exponent *λ* into the original radiation model, borrowed from its counterpart the so-called gravity model, to enable the applicability of the radiation model to various mobility systems, despite of the diversities of human mobility at different spatial scales. Additionally, we propose four variations of the model based on combinations of searching direction and trip OD constraint, which indicate the driving force of individuals’ movements and the systematic limits of mobility flux. These variations can further improve model results in different scenarios and mobility systems. The last modification lies in the thermodynamic limit assumption for the original radiation model. For a finite system, we revise the model with correct normalization factors, which are appropriate extensions of previous work [13]. Our empirical analyses in different countries and cities confirm that these modifications are necessary and effective. The generalized radiation model not only reliably reproduces the actual characteristics of human movements observed in real data, but also brings itself and the gravity model in a consistent form in terms of model parameters. These results open new directions for extending the radiation model to practical systems and applications from urban planning, traffic engineering to mobile location-based services.

## Results

### Generalized Radiation Models

As proposed in Simini’s original work [10], the source and destination of a commuting is determined by a process of job selection that consists of two separate steps: (1) job seeking, which assumes a proportional relation between the number of employment opportunities in each location and the resident population; and (2) job selecting, whose criteria is to choose the closest job with a benefit higher than the best offer available in the home location. Mathematically, accumulating the probability of all resident population in each location yields the daily commuting fluxes between different locations as (refer to Table 1 for detailed parameter description)

$$<T_{ij}>\equiv T_{i}\frac{m_{i}n_{j}}{\left(m_{i}+s_{ij}\right)\left(m_{i}+n_{j}+s_{ij}\right)}$$

**Table 1:** The generalized radiation models with scaling exponent, searching direction and trip OD constraint. From left to right, we generalize the model by categorizing it as intervention-based and competition-based with regard to the motivations of individual human travels. From top to bottom, we further generalize the model by adding trip OD constraints into the model, and obtain the production-constrained and the attraction-constrained forms of the radiation model.

**Generalized Radiation Model**		
	Intervention	Competition
Production	$\kappa \cdot \frac{T_{i}}{\sum_{k\neq i}^{N}{\left\{\frac{m_{i}n_{k}}{\left(m_{i}+s_{ik}\right)\left(m_{i}+n_{k}+s_{ik}\right)}\right\}}^{\lambda }}\cdot {\left\{\frac{m_{i}n_{j}}{\left(m_{i}+s_{ij}\right)\left(m_{i}+n_{j}+s_{ij}\right)}\right\}}^{\lambda }$	$\kappa \cdot \frac{T_{i}}{\sum_{k\neq i}^{N}{\left\{\frac{n_{k}m_{i}}{\left(n_{k}+s_{ki}\right)\left(n_{k}+m_{i}+s_{ki}\right)}\right\}}^{\lambda }}\cdot {\left\{\frac{n_{j}m_{i}}{\left(n_{j}+s_{ji}\right)\left(n_{j}+m_{i}+s_{ji}\right)}\right\}}^{\lambda }$
Attraction	$\kappa \cdot \frac{T_{j}}{\sum_{k\neq j}^{N}{\left\{\frac{m_{k}n_{j}}{\left(m_{k}+s_{kj}\right)\left(m_{k}+n_{j}+s_{kj}\right)}\right\}}^{\lambda }}\cdot {\left\{\frac{m_{i}n_{j}}{\left(m_{i}+s_{ij}\right)\left(m_{i}+n_{j}+s_{ij}\right)}\right\}}^{\lambda }$	$\kappa \cdot \frac{T_{j}}{\sum_{k\neq j}^{N}{\left\{\frac{n_{j}m_{k}}{\left(n_{j}+s_{jk}\right)\left(n_{j}+m_{k}+s_{jk}\right)}\right\}}^{\lambda }}\cdot {\left\{\frac{n_{j}m_{i}}{\left(n_{j}+s_{ji}\right)\left(n_{j}+m_{i}+s_{ji}\right)}\right\}}^{\lambda }$
**Parameter Description**		
*T*_*i*_	Total number of trips departing from location *i*		
*T*_*j*_	Total number of trips terminating at location *j*		
*m*_*i*_	Total population (or trips) at location *i*		
*n*_*j*_	Total population (or trips) at location *j*		
*s*_*ij*_	Total population in the circle of radius *r*_*ij*_ centred at *i* (excluding the source and destination population)		
*s*_*ji*_	Total population in the circle of radius *r*_*ji*_ centred at *j* (excluding the source and destination population)		
*κ*	Normalization constant of total number of trips		
*λ*	Scaling exponent of spatial extents		
*N*	Total number of locations		

Enlightened by Wilson’s works on the generalization of the classical “gravity model” [15, 16], we developed a class of generalized radiation models, listed in Table 1 (see S2 Text for details of model deriviation). Notice that the original radiation model is derived from a stochastic decision-making process of individual’s destination selection, which defines the probability for a transition from location *i* to location *j* (see S2 Text). We first re-scale this transition probability with an exponent *λ*, which is mobility system dependent (see S3 Text for parameter determination). We argue that this scaling exponent is a meaningful indicator of the economies of agglomeration and can capture the “scale-free” property of distance-decay effect as implied in the gravity-based models [17]. It is worth noting immediately that the original radiation model is benefit-motived, or in other words, intervention-based. Before an individual selects a destination *j*, she/he will assess the benefit of each location’s opportunities. The more opportunities *n*_*j*_ and the less interventions *s*_*ij*_ a location *j* has, the higher the benefit it offers and the higher the chance of it being chosen is. In this research, we reverse this process and obtain the competition-based radiation model (refer to S2 Text). As a result, a transition from location *i* to location *j* is driven by the demands emit by the destination accordingly. The more supplies *m*_*i*_ and the less competitions *s*_*ji*_ a location *i* has, the higher the chance of it being chosen will be.

Noticing that the original radiation model is in an unconstrained form and derived from an infinite system, we further correct the predicted flux, *T*_*ij*_, to ensure the origin and destination constraints on fluxes are met in finite systems. More importantly, the density of the observed mobility network is usually very sparse (see S1 Table). Consequently, the original radiation model systematically underestimates the travel fluxes between observed location pairs. To address this issue, we therefore explicitly adjust flux *T*_*ij*_ with a normalization factor *κ* to ensure the total predicated fluxes and the total observed fluxes are matched. Eventually, we obtain four variants of the generalized radiation model: (1) Production-constrained Intervention-based Radiation (**PIR**) model, (2) Production-constrained Competition-based Radiation (**PCR**) model, (3) Attraction-constrained Intervention-based Radiation (**AIR**) model, and (4) Attraction-constrained Competition-based Radiation (**ACR**) model. Each of these generalized radiation models has a scaling exponent *λ* and a normalization factor *κ*, with which they can be applied to diverse mobility systems.

### Fitting and Predicting Mobility Fluxes

In the previous section, we introduced a new family of generalized radiation models. Since the new models are with parameters *λ* and *κ*, empirically observed mobility data are needed for parameter estimation. Here we describe the method that we used to determine the best values for the scaling exponent *λ* and the normalization factor *κ*. As aforementioned, the normalization constant *κ* can be directly obtained by comparing the total predicated fluxes and the total observed fluxes. Thus, we put the emphasis on the determination of the scaling exponent *λ*.

**Fig 1:**
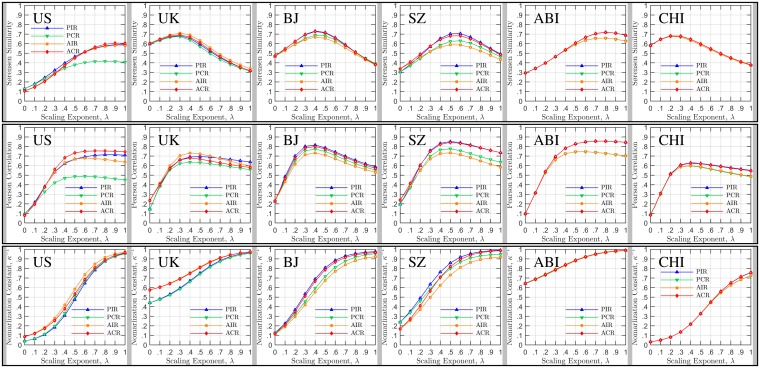
Parameter estimation for the generalized radiation model. In the top panels, we vary the scaling exponent λ for each model, and compute the Sørensen-Dice similarity between the model output and the real data. The λ yielding the peak similarity value is the estimated parameter of the adopted model. In the middle panels, the Pearson Correlation Coefficient (PCC) is calculated between the model output and the real data. In the bottom panels, the correct normalization factor κ is derived for models with different scaling exponents. Note that the estimated scaling exponent λ is 1.0, 0.3, 0.4, 0.6, 0.8, 0.2 for US, UK, Beijing, Shenzhen, Abidjan and Chicago, respectively.

To fit the model with empirical mobility data, a measure of goodness of fit is firstly needed. As stated in existing literature, the *R* ^2^ value is not a well-received evaluation index, especially in the case of the radiation model. In this paper, we hence perform a fluctuations analysis based on the Sørensen-Dice coefficient [18, 19] (see S2 Text) to quantify the degree of similarity with real observations. If there is no match between the empirical data and the model output this coefficient equals to 0, whereas it is 1 for a complete match. By fitting the generalized radiation models with incremental exponent *λ* = [0 : 0.1 : 1] to the empirical mobility data (more details are presented in S3 Text), we choose the model yielding the best goodness of fit, Sørensen, as the adopted predicting model. As shown in Fig 1, the scaling exponent *λ* is system sensitive, indicating that the original radiation model is not as universal as expected in existing literature [10]. In the analysis, we also find a strong and negative correlation between parameters *κ* and *λ*. As the scaling exponent *λ* increases, the normalization factor *κ* decreases, with a baseline largely relying upon the density of each empirical mobility network.

**Fig 2:**
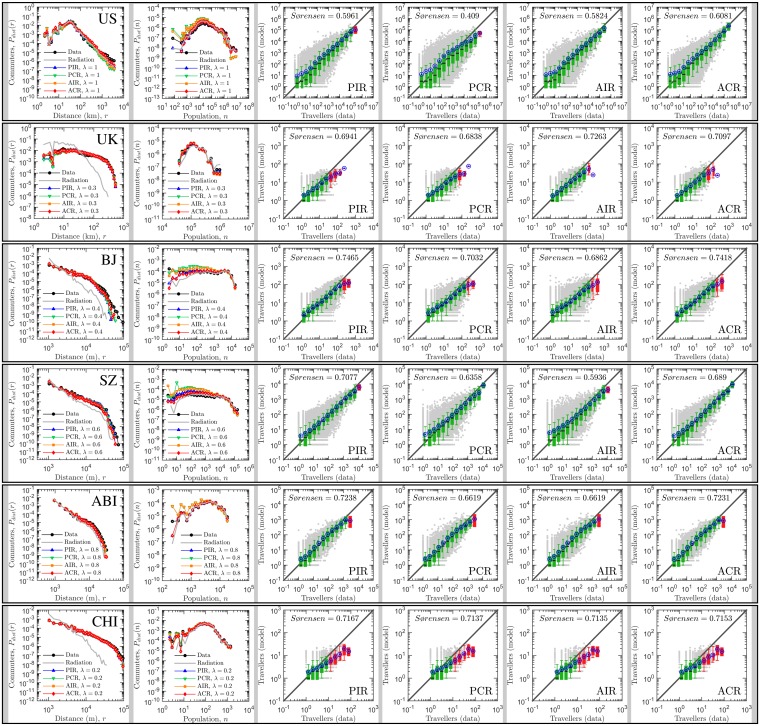
Comparing the predictions of the generalized radiation model and the real data. In the first column, these panels display the overall travel distance distribution reported in the empirical data and the fitted models. It reveals the probability of a trip between two locations that are at distance *r* (in km) from each other, *P*_dist_(*r*). These distributions are generally collapsed with each other, indicating the predictions of the model are acceptable. In the second column, the panels display the distributions of fluxes associated with given population at destination or origin. It denotes the probability of a trip from or towards a location with population *n*, *P*_*dist*_(*n*). Again, agreements between the model output and the real data are observed. In columns 3 to 6, these panels compares the observed flux, *T* ^*data*^, with the predicted flux, *T* ^*model*^, for each pair of *i*, *j* counties where real travel flux exists. Note that gray points are scatter plot for each pair of locations. A box is colored green if the diagonal line lies between the 5th and the 95th percentiles in that bin and is red otherwise. The black circles correspond to the mean number of predicted travelers in that bin. In general, the generalized radiation model predicts travel fluxes in well agreement with the real data, except for flows with large volumes.

To validate the power of the generalized radiation models, we conducted evaluations with six different real-world datasets: human daily travel data from two countries and four cities collected by census survey, GPS, mobile phone and traditional household surveys (see details in S1 Text). Note that the corresponding human mobility systems are of different spatial scales and transport modes, aiming to test the universality of the generalized radiation model. As shown in Fig 2, for all studied cases the generalized radiation model outputs plausible results and exhibits relatively high index values (Sørenson≈0.7), indicating that the generalized radiation model effectively captures the underlying mechanism that drives human movement across different scales and systems.

### Model Validation and Comparison

We compare three statistical measures between the model output and the real data for model validation. The first measure is the overall travel distance distribution predicted by the generalized radiation models based on real data where these data is available. The travel distance distribution is widely taken as an important statistical property to capture human mobility behaviors and reflect a city’s economic efficiency. We measure the probability *P*_*dist*_(*r*) of a trip between locations at distance *r* (see the first column of Fig 2) and run the two-sample Kolmogorov-Smirnov test to verify whether the distributions of the model output and the real data are generated by an identical distribution at the 5% significance level. As shown in Table 2, the distance distribution of each model output is statistically identical to its counterpart of the real data set (i.e., *p*-values are larger than 0.9 for most of our case studies), indicating that the generalized radiation models can predict travel distance distribution with good fidelity. In contrast, the original radiation model failed to reproduce the distance distributions, except for US which has the scaling exponent *λ* = 1 (see the first column of Fig 2). This provides strong evidence for the validity of the universality of our generalized radiation models. Under closer scrutiny, we further observe the shapes of the distance distributions vary from case to case, suggesting that human movements do not exhibit universal patterns across cities as stated in existing literature [20, 21]. Except for US, the distribution of travel distance cannot be approximated with a power-law distribution in the tail [22]. This observation confirms existing findings in the literature [11, 23, 24], which regard the density of place and population as a decisive factor in human mobility.

**Table 2:** Agreements between the travel fluxes predicted by the generalized radiation model and the travel fluxes observed in the real data. We conduct the two-sample Kolmogorov-Smirnov test to verify whether the distributions of the model and the data are from an identical distribution at the 5% significance level in terms of overall travel distance distribution and travels towards (or from) given population. Values in red indicates non-agreements between the model prediction and the real data.

*p*-value at the 5% significance level										
	Distance					Population				
	Radiation	PIR	PCR	AIR	ACR	Radiation	PIR	PCR	AIR	ACR
US	0.9713	0.9713	0.9713	0.9713	0.9713	0.4225	0.4225	0.0619	0.1078	0.5953
UK	0.0004	0.9354	0.9354	0.4519	0.4519	0.8899	0.9983	0.8899	0.8899	0.8899
Beijing	0.0222	0.8107	0.9570	0.9570	0.9570	0.0026	0.3456	**0.0000**	**0.0001**	0.1297
Shenzhen	0.2179	0.9509	0.9982	0.9982	0.9982	0.4141	**0.0001**	**0.0000**	**0.0000**	0.0520
Abidjan	0.5372	0.9970	0.9970	0.9970	0.9970	0.9998	0.9563	0.4622	0.4622	0.9563
Chicago	0.0019	0.9672	0.9672	0.9672	0.9672	0.3003	0.9168	0.9168	0.9168	0.9168

The second measure adopted is the probability *P*_*dist*_(*n*) of trips towards a destination (for production-constrained models) or from a origin (for attraction-constrained models) with population *n*. Note that *P*_*dist*_(*n*) is a key quantity for measuring the accuracy of singly-constrained mobility models, in that singly-constrained models cannot ensure the agreement between the predicted travel to a location and the real travel to the same location. We then run the two-sample Kolmogorov-Smirnov test and verify whether the population distributions of the model and the real data are generated from an identical distribution at the 5% significance level. Out of the 24 model outcomes (i.e., four models for six different data sets), 19 are identical with their corresponding real data (see Table 2). The distribution curves in the second column of Fig 2 also confirm that the generalized radiation models can predict the travel population distribution in the real data with high accuracy, whereas the original radiation model has poorer performance. Additionally, we find that the generalized model better predicts empirical observations with large population (at the tail) compared with observations with small population (at the head) in these figures, implying the high fluctuations of low volume travel fluxes. Here again, no common trends of the distributions of the travel fluxes with population at destination (or origin) are observed. This fact indicates that the spatial patterns of population distribution in our case studied areas are different, suggesting a potential relationship between spatial heterogeneity and travel behaviors. For instance, within a very dense metropolis, like Beijing and Shenzhen, there is a relatively higher expectation of short-range movements. This analysis also well exhibits the diversity of our case studied mobility data.

Last, we implement a more detailed measure of a model’s prediction ability for mobility patterns in terms of the coincidence between the predicted and real travel fluxes between all pairs of locations produced by the candidate model in comparison with real observations. As shown in columns 4 – 7 of Fig 2, we find that, except for the case of Abidjan, the average fluxes predicted by the radiation model highly coincide with the real fluxes, demonstrating a reasonable agreement with real observations. Note that the Box-Whisker plot method used here cannot allow an explicit comparison for distinguishing the performance of different candidate models. In this regard, we adopt the Sørensen-Dice coefficient to quantify the degree of similarity between the model predictions and the real observations as discussed in the previous section. For comparison, we also exploit the Pearson Correlation Coefficient (PCC) to measure the consistence between the predicted mobility flow and the actual mobility flow for origin-destination pairs that have actual flows. Note that the PCC is computed not with logarithmic travel flux values but actual travel flux values (see S3 Text) and it is a rigorous comparison criterion that tests the strength of linear relationship between model and data. The higher the PCC is, the higher the ability of the model to predict the travel flow values for an individual origin-destination pair will be. As shown in Fig 1, our model exhibits Sørensen-Dice index values as high as 0.7. Whereas, outcomes of the original radiation model are 0.59, 0.32, 0.38, 0.48, 0.65, 0.37 for US, UK, Beijing, Shenzhen, Abidjan and Chicago, respectively. Note that similar results were also reported in existing literature [10, 12]. This result indicates that the generalized model outperforms the original radiation model, and captures the underlying mechanism that drives human movements more appropriately. In Fig 1, it is also evidently to notice that the PCC index is at a high level for all case studies. However, there is no significant peak value in the distribution of the PCC index along with scaling exponent *λ*. This finding confirms that our principle of determining the best model by the Sørensen-Dice index instead of the PCC index is effective.

**Fig 3:**
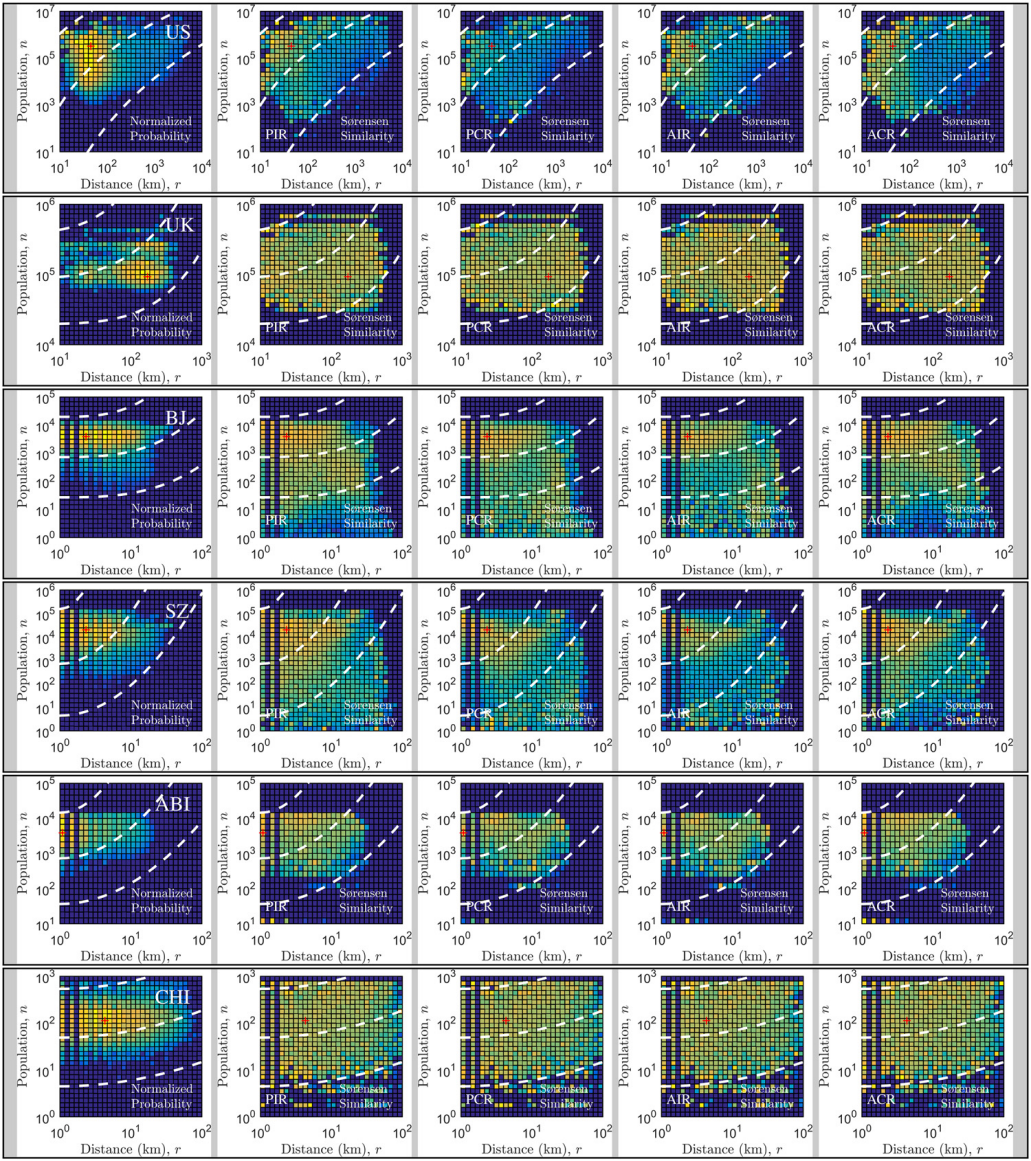
Model error as a function of spatial distance and population at destination. In the left panels, we show the normalized probability of traveling associated with given distance and population observed in the real data. The lighter the color is, the higher the probability is, which implies where travel fluxes in the mobility network are concentrated. In other panels, we show the Sørensen-Dice coefficient of the generalized radiation models. Note that the coefficient is in the range of 0 and 1 for all the case studies, and is assigned the same color for the same value. The lighter the color is, the higher the similarity between the model output and the real data is, which indicates the power of the predicting models over different distances and origin or destination population. The dashed line shows the trend of travel flux and the similarity index distributed along with distance and population.

In Fig 3, we further present the analysis of model error as a function of two sensitive parameters, the travel distance *r* and the destination population *n*. In the left panels (first column) of the figure, we show the normalized probability of travel population in different locations in the phase space made up by distance, population at destination, and empirical flows. We see that these distributions show apparently distinct patterns. For instance, in the US commuting data most travel fluxes are between locations with short distance and large destination population. By comparison, in the UK commuting data most travel fluxes are observed covering long distance (around 45 km) and towards moderate destination population. It is possible to see the correlations between the performances of the generalized models and the mobility systems with distinct flux patterns spanning travel distances and destination population. We find that the higher the proportion of travel population with long travel distance is, the smaller the scaling exponent *λ* of the best model is. In more details, for mobility systems whose travel population are short-distance and large-destination-population dominant, like US, Abidjan and Shenzhen, the generalized radiation model poorly estimates the flows for large distance and small destination population. As a comparison, for mobility systems like UK, Beijing and Chicago whose travel population are long-distance and moderate-destination-population dominant, the generalized radiation model better estimates the flows over the entire phase space. From these panels, we also observe that the generalized radiation model performs more plausibly for travel fluxes where the majority of flows are concentrated.

## Discussion

In this article, we generalized the radiation model and performed empirical validation of the model with human movements in mobility systems with diverse characteristics. Inspired by Stouffer’s theory of intervening opportunities, the original radiation model offers a plausible explanation to certain observed mobility patterns. However, despite having the advantage of being parameter-free and performing well at large spatial scale, it cannot offer satisfactory predictions of mobility patterns at different scale and for different mobility data. The problem lies in its ignorance of properties of the mobility networks and motivations of human’s traveling. To consolidate its nature of universality, we improved the radiation model as an analog to the generalization of the classic “gravity model”. By imposing the appropriate scaling exponent *λ*, normalization factor *κ* and system constraints including searching direction and trip OD constraint, the generalized radiation model effectively captured real human movements both at both macroscopic and microscopic scales. Our generalized radiation model also outperformed alternative common models, like gravity models and intervening opportunities models, in various empirical analyses.

As discussed above, the radiation model assumes that individuals prefer to traveling towards a farther location than a nearby location if there are more opportunities and benefits. This assumption results in the parameter-free property, but meanwhile scarifies the flexibility and adaptability of the model. In particular, the original radiation model was ill-designed for predicting short-range travels within cities and thus performs relatively poor for intra-city mobility [25, 26]. The generalized radiation model can effectively overcome this problem by imposing system-specific parameters that can be derived from fitting the model to empirical mobility networks in parallel with the classic gravity model. We observe that performances of the generalized models largely rely on mobility patterns spanning different travel distances and destination population. A higher proportion of travel population with long travel distance generally indicates a smaller scaling exponent *λ* for the best model. More specifically, if travel population are short-range and large-destination-population dominant, the proposed model poorly predicts the flows for large distance and small destination population. Whereas, when travel population are long-range and moderate-destination-population dominant, it better estimates the flows over the entire phase space. Insofar to our best of knowledge, our model presently offers the best prediction of mobility patterns at different scales, significantly deepening our understanding of human mobility and demonstrating the universal predictability of mobility patterns. Moreover, the richness of the family of the generalized radiation models naturally opens up new research directions, such as the identification of the needs and motivations driving human movements. For instance, as implied by the analytic results in Fig 2, the mobility networks of US and Shenzhen are, with a slightly higher level of statistical significance, competition-based and attraction-constrained. Meanwhile, from the fluctuation analysis in Fig 3, it emerges that there is a consistent portion of the travel distance and the destination population phase space where the radiation model gives plausible estimates in terms of the Sørensen-Dice coefficient. It implies the possibility of tweaking the form and the scaling exponent of the model to yield plausible predications meeting specific requirements on travel distance and destination population. This flexibility indicates that we largely improved the reliability and universality of the radiation model.

We have also compared the predicted travel flows by the gravity model, the original radiation model, the intervening opportunities model and our generalized model within our 6 case studies areas. In most cases, our parametrized models can yield better or equal predictive accuracy than the competitive models. Note that performances of the gravity-based model for the 6 cities were reported in [12] and they are significantly worse than those of the generalized radiation models. Although these models have different hypotheses, they share an underlying mechanism that quantifies the distance-decay effect. This suggests that we may further improve the predictive ability of human mobility models by adjusting the way of quantifying the distance [27] and the population [13] of the underlying mobility system. Another direction of improvements lies in the structure of the predicted mobility network against with the real data. We find that though the generalized radiation model performs great in terms of distance and population measures, its ability of reproducing the community structure of the mobility network is limited. The overlaps between the model and the real data are highly random along with the scaling exponent *λ* and span a large range in terms of the adjusted Rand index [28]. For the sake of simplicity, the analytic results on the comparison of community structures are excluded in the article. More comprehensive model validations will be conducted in the future works.

## Supporting Information

S1 Text Data Description. (PDF)

S1 Table Statistical Characteristics of the Mobility Datasets in Our Case Studies. (PDF)

S2 Text Derivation of the Generalized Radiation Model. (PDF)

S3 Text Selection of the Scaling Exponent *λ*. (PDF)

S4 Text Data Accessibility. (PDF)
