# Supplementary material for: A Generalized Radiation Model for Human Mobility: Spatial Scale, Searching Direction and Trip Constraint
Source: PLoS One. 2015 Nov 24;10(11):e0143500. doi: 10.1371/journal.pone.0143500 (PMC4657960; doi:10.1371/journal.pone.0143500)
Supplement: S1 Text — (PDF) [file pone.0143500.s001.pdf]

S1 Text. Data Description.

– US commuting – The dataset is collected by US Census 2000 on the home and workplace of each sampled individual at the county level for residents of the 50 states and the District of Columbia (DC) (available online at `http://www.census.gov/population/www/cen2000/commuting/index.html`). In this article, we only analyzed the commuting flow with the US Continent (without Alaska, Hawaii and offshore islands).

– UK commuting – The dataset contains commuting patterns for England and Wales at Local Authority (LA) level resolution from the 2001 Census Journey to Work data (available at `http://www.ons.gov.uk/ons/index.html`). In the dataset, the origin and destination wards of each surveyed commuters are recorded.

– BEIJING and SHENZHEN– The datasets contain the travel records of taxi passengers in Beijing and Shenzhen during a week respectively (shared online at `http://sss.bnu.edu.cn/~wenxuw/data_set.htm`). Coordinates and time of passenger pick-up and drop-off are explicitly recorded in the data. It is well-received that taxi OD data, being a small subset of the population movements, can capture the travel pattern of urban residents to some extent.

– ABIDJAN – The dataset captures mobile phone subscribers' movements in Abidjan, the biggest city of Ivory Coast, during a two-week observation period. Each record is anonymized, and logs a phone call or SMS exchange between two mobile phone users in Ivory Coast.

– CHICAGO – The dataset comes from the household travel tracker survey in Chicago Metropolitan areas between January 2007 to February 2008 (available online at `http://www.cmap.illinois.gov/travel-tracker-survey/`). The coordinates of the trip's origin and destination of surveyed householders are collected.

Statistical characteristics of these mobility datasets are presented in **S1 Table**.
