# Supplementary material for: A Generalized Radiation Model for Human Mobility: Spatial Scale, Searching Direction and Trip Constraint
Source: PLoS One. 2015 Nov 24;10(11):e0143500. doi: 10.1371/journal.pone.0143500 (PMC4657960; doi:10.1371/journal.pone.0143500)
Supplement: S1 Table — (PDF) [file pone.0143500.s002.pdf]

S1 Table. Statistical Characteristics of the Mobility Datasets in Our Case Studies.

	Node	Edge	Density	Population	Traveller	Modularity	Community
US	3,062	159,707	0.017	[65, 9538191]	34,010,098	0.8921	47
UK	346	45,487	0.381	[34700, 1073000]	135,747	0.2973	4
Beijing	3,025	194,415	0.021	[1, 17974]	1,048,981	0.3982	11
Shenzhen	1,634	117,238	0.044	[1, 192003]	2,337,286	0.4621	14
Abidjan	219	25,493	0.534	[20, 17731]	519,710	0.6166	6
Chicago	1,406	35,795	0.018	[1, 1299]	72,063	0.5847	8

S2 Table. Statistical characteristics of the mobility datasets in our case studies. We collect trip OD matrices in two countries (US and UK) and four cities (Beijing and Shenzhen in Asia, Abidjan in Africa and Chicago in North America). The mobility networks are with significantly distinct properties. The number of nodes (locations) varies from hundreds to thousands. And edges in each network are different in terms of both total numbers and density (defined as the probability of movements being observed between two arbitrary locations). The modularity of each mobility network well indicates if the travel flows are concentrated in certain locations.
