# Supplementary material for: A Generalized Radiation Model for Human Mobility: Spatial Scale, Searching Direction and Trip Constraint
Source: PLoS One. 2015 Nov 24;10(11):e0143500. doi: 10.1371/journal.pone.0143500 (PMC4657960; doi:10.1371/journal.pone.0143500)
Supplement: S2 Text — (PDF) [file pone.0143500.s003.pdf]

S2 Text. Derivation of the Generalized Radiation Model

Here we introduce a modeling framework of generalizing the radiation model. As proposed in the original work [1], the source and destination of a commuting is determined by job selection that consists of two steps:

Step one, job seeking. It assumes that the number of employment opportunities in each location is proportional to the resident population, n. If there is one job opening for every n_{jobs} individuals, each county with population n will be assigned n/n_{jobs} random benefits, z_1, z_2, ..., $z_{[n/n_{jobs}]}$. It ensures that the larger a location's population is, the more employment opportunities it offers.

Step two, job selecting. The criteria is to choose the closest job with a benefit z higher than the best offer available in his/her home location. Quantitatively, the probability of traveling from location i to j can be computed as (refer to Table 1 in the main text for detailed parameter description)

$$P(1|m_i, n_j, s_{ij}) = \frac{m_i n_j}{(m_i + s_{ij})(m_i + n_j + s_{ij})} \tag{1}$$

Accumulating the probability of all resident population in each location, we can obtain the daily commuting fluxes across the country as

$$< T_{ij} >\equiv T_i \frac{m_i n_j}{(m_i + s_{ij})(m_i + n_j + s_{ij})} \tag{2}$$

Obviously, this original radiation model is intervention-based and we can directly reverse aforementioned assumptions into a competition-based form as:

Step one, an individual seeks employement with skill level of z from all locations, including his/her home location. We assumes that the number of employement supplies in each county is proportional to the resident population, n. Thus, each location with population n is assigned $n/n_{workers}$ random benefits, z_1, z_2, ..., $z_{[n/n_{workers}]}$. Like the original model, the larger a county's population is, the more employement supplies it offers.

Step two, the individual chooses the closest supply to his/her home, whose skill level z are higher than the best offer available in his/her home location. It immediately comes that the competition-based radiation model is exactly the inverse process of the intervention-based radiation model. It therefore gives the relations that:

$$P^{competition}(1|n_j, m_i, s_{ij}) \equiv P^{intervention}(1|m_i, n_j, s_{ij}) \tag{3}$$

Together with the scaling exponent and the constraints of trip production and attraction, we immediately obtain four variants of the original radiation model as: (1) Production-constrained Intervention-based Radiation model, (2) Production-constrained Competition-based Radiation model, (3) Attraction-constrained Intervention-based Radiation model, and (4) Attraction-constrained Competition-based Radiation model.

References

[1] Simini F, González MC, Maritan A, Barabási AL. A universal model for mobility and migration patterns. Nature. 2012;484(7392):96–100.
