# Supplementary material for: A Generalized Radiation Model for Human Mobility: Spatial Scale, Searching Direction and Trip Constraint
Source: PLoS One. 2015 Nov 24;10(11):e0143500. doi: 10.1371/journal.pone.0143500 (PMC4657960; doi:10.1371/journal.pone.0143500)
Supplement: S3 Text — (PDF) [file pone.0143500.s004.pdf]

S3 Text. Selection of the Scaling Exponent λ

Here we describe the method that we used to find the best values for parameters of the generalized radiation model. We define the error functions as negative of the Sørensen-Dice correlation coefficient

$$Sørensen \equiv \frac{2\sum_{i,j} min(T_{ij}^{data}, T_{ij}^{model})}{\sum_{i,j} T_{ij}^{data} + \sum_{i,j} T_{ij}^{model}} \tag{1}$$

and the Pearson correlation coefficient

$$\rho \equiv \frac{\sum_{i,j}(T_{ij}^{data} - T_{mean}^{data})(T_{ij}^{model} - T_{mean}^{model})}{\sqrt{\sum_{i,j}(T_{ij}^{data} - T_{mean}^{data})^2}\sqrt{\sum_{ij}(T_{ij}^{model} - T_{mean}^{model})^2}} \tag{2}$$

By fitting the generalized radiation models with incremental exponent $\lambda = [0 : 0.1 : 1]$ to the empirical mobility data, we select the model yielding the best goodness of fit, *Sørensen*, as the adopted predicting model, since that it is harder to tell the peak value of the distribution of ρ (see Fig 1 in the main text). For all studied cases, the generalized radiation model produces plausible results and exhibits relatively high index values (i.e., approximately 0.7). It suggests that the generalized models capture the underlying mechanism driving human movement across different scales and systems effectively.
