# Supplementary material for: A Generalized Radiation Model for Human Mobility: Spatial Scale, Searching Direction and Trip Constraint
Source: PLoS One. 2015 Nov 24;10(11):e0143500. doi: 10.1371/journal.pone.0143500 (PMC4657960; doi:10.1371/journal.pone.0143500)
Supplement: S4 Text — (PDF) [file pone.0143500.s005.pdf]

S4 Text. Data Accessibility

All datasets (including both the mobility networks and the population distributions) used by the proposed models are available here.
